# The Anatomy of the Stylohyoid Chain: A Systematic Review with Meta-Analysis

**DOI:** 10.3390/diagnostics15070925

**Published:** 2025-04-03

**Authors:** George Triantafyllou, Ioannis Paschopoulos, Fabrice Duparc, George Tsakotos, Panagiotis Papadopoulos-Manolarakis, Maria Piagkou

**Affiliations:** 1 Department of Anatomy, Faculty of Health Sciences, School of Medicine, National and Kapodistrian University of Athens, 11527 Athens, Greece; georgerose406@gmail.com (G.T.); jonhpascho@gmail.com (I.P.); gtsakotos@gmail.com (G.T.); p.papado89@gmail.com (P.P.-M.); 2 Department of Anatomy, Faculty of Medicine-Pharmacy, University of Rouen-Normandy, 76000 Rouen, France; fabrice.duparc@univ-rouen.fr; 3 Department of Neurosurgery, General Hospital of Nikaia-Piraeus, 18454 Athens, Greece

**Keywords:** temporal bone styloid process, stylohyoid chain, elongated styloid process, variation, clinical anatomy, evidence-based anatomy

## Abstract

**Background**: The temporal bone’s styloid process (SP) is an important structure that extends from the skull base to the parapharyngeal space. The stylohyoid ligament (SHL) attaches it to the hyoid bone. The SP and SHL are considered the stylohyoid chain (SHC) components. The SP’s close relationship with vital head and neck structures has important clinical implications. Specifically, SP and SHC variants are linked with clinical conditions. Therefore, adequate knowledge of these variations is of paramount importance. **Methods**: Using the latest guidelines, a systematic literature review was performed in four online databases (PubMed, Google Scholar, Scopus, and Web of Science) to identify studies referring to the SP’s typical anatomy and possible SHC morphological variants. The meta-analysis was conducted using R programming software to calculate the prevalence of typical anatomy and possible variants and the pooled mean length of the SP. **Results**: A total of 104 studies were included, with a total sample of 136,010 heminecks. The typical SP (under 30 mm) was estimated to have a pooled prevalence of 74.97%. SP elongation was observed in 25.03%. The subgroup analysis identified significant differences based on the study type, with computed tomography (CT) studies having the highest pooled prevalence. The SP length was calculated to have a pooled mean of 28.91 mm. For SHC ossification, the pseudo-articulated type was identified to have a pooled prevalence of 4.39%, and that of the segmented type was detected to be 3.89%. The geographical distribution and study type affected the estimated pooled prevalence. **Conclusions**: The current evidence-based systematic review with meta-analysis investigated the SHC’s typical anatomy and possible variants. The elongated SP pooled prevalence of 25.03% indicates that it is not a rare variant, and CT is the optimal method to investigate such a variant. These details demonstrated by the current meta-analysis could be of importance for clinicians.

## 1. Introduction

Clinical anatomy of the head and neck region is important for anatomists, radiologists, and surgeons due to the several pathologies and procedures that can develop in the area [1,2,3,4,5,6].

The styloid process (SP) is a long, slender, cylindrical bony projection from the petrous part of the temporal bone. The stylohyoid chain (SHC) consists of the SP, the stylohyoid ligament (SHL), and the lesser horns of the hyoid bone (HB and LH). The ossification of Reichert’s cartilage forms the SP; the section of this cartilage between the SP and the LH of the HB creates the SHL and corresponds to the cartilaginous segment known as the ceratohyal segment [7]. In human development, Rodríguez-Vázquez et al. [8] did not observe the previously described segmentation of the stylohyoid apparatus, including the tympanohyal, stylohyal, ceratohyal, and hypohyal segments [9]. They observed only the cranial segment of Reichert’s cartilage corresponding to the stylohyal segment, as well as the segment corresponding to the lesser horn or hypohyal segment [8].

A few muscles (styloglossus, stylopharyngeus, and stylohyoid) and ligaments (stylohyoid and stylomandibular) are attached to the SP and perform several movements. The SP extends posteriorly to the tympanic plate and projects anteroinferiorly into the parapharyngeal space. The prestyloid compartment contains the parotid gland, the facial and lingual nerve, and the external carotid artery (ECA). The retrostyloid compartment contains the internal carotid artery (ICA), the internal jugular vein, the sympathetic chain, and the extracranial segments of the IX–XII cranial nerves [10,11].

SHC variants, including SP variants, exhibit significant variability in both SP length and SHC ossification, which may encompass two to five segments [12]. SP length has been the subject of extensive research. Eagle [13] was the first to report the clinical importance of SP length. Typically, it measures between 25 and 30 mm; however, it can be frequently observed as elongated (over 30 mm) [1,12,13]. The clinical significance of an elongated SP has been described as stylohyoid or Eagle syndrome [1,14]. Another infrequent condition implicating the SP is its fracture, which is related to post-traumatic pain and misdiagnosed lateral cervical pain [14].

According to Rodriguez-Vasquez et al.’s [15] morphogenetic study, variability in the form and length of the cranial segment of Reichert’s cartilage gives rise to an SP of variable length that can be inclined, as observed by Baddour et al. [16]. Further research was carried out by Lengele and Dhem [17], which showed that both long and short SPs presented the same characteristics as calcified cartilage. The position of the angulated inferior end of the cranial or styloid segment of Reichert’s cartilage may explain the most frequent symptomatology associated with Eagle’s syndrome. Rodríguez-Vázquez et al. [15] considered that dysphagia and the sensation of a foreign body in the throat could be caused by the tip of the angulated end sometimes being very close to the pharyngeal wall. This is why Frommer [18] observed that the SP direction and curvature were more important than its length. A sore throat and pain around the area of distribution of the glossopharyngeal nerve [18], and even possible alterations in taste [16,19], could be explained by the close association we have demonstrated between the nerve and Reichert’s cartilage. According to Graf [20], while swallowing, the glossopharyngeal nerve could be pushed against the osseous spicule and be stimulated, producing paroxysmal pain.

We read with great interest a previous meta-analysis that explored the pooled prevalence of an elongated SP in imaging studies [21]. Therefore, in the current systematic review with meta-analysis, we aimed to examine both typical and elongated SPs and the SHC ossification status across imaging and osteological studies.

## 2. Materials and Methods

The systematic review with meta-analysis was performed following the methods of the Evidence-Based Anatomy Workgroup for anatomical meta-analysis [22] and the PRISMA 2020 guidelines [23], similar to previous studies [24,25]. This study’s protocol was registered in the PROSPERO database (CRD420250656646).

A literature search was performed on the online databases PubMed, Google Scholar, Scopus, and Web of Science during August 2024. The following terms were used in several combinations: “styloid process”, “elongated styloid process”, “stylohyoid chain”, “variation”, “anatomical study”, “cadaveric study”, “osteological study”, “radiologic study”, “computed tomography study”, and “imaging study”. Moreover, the references of all included articles were evaluated, the grey literature was investigated, and a manual search of significant anatomical journals (Annals of Anatomy, Clinical Anatomy, Journal of Anatomy, Anatomical Record, Surgical and Radiological Anatomy, Folia Morphologica, European Journal of Anatomy, Anatomical Science International, and Anatomy and Cell Biology) was performed. The inclusion criteria were studies reporting the prevalence of typical and elongated SPs and their ossification status. Case reports, conference abstracts, animal studies, and studies that reported irrelevant or insufficient data were excluded. Additionally, we excluded studies that included only pathological populations (e.g., Eagle syndrome patients).

Two independent reviewers (GTr and IP) performed the literature search and extracted the data into Microsoft Excel sheets. The results were compared, and the other authors resolved potential differences. The Anatomical Quality Assurance (AQUA) tool, created by the Evidence-Based Anatomy Workgroup for anatomical reviews [26], was used to evaluate each article’s risk of bias (Table S1).

Statistical meta-analysis was conducted with the open-source R programming language and RStudio software (version 4.3.2) using the “meta” and “metafor” packages. The pooled prevalence was calculated using the inverse variance and random-effects models. The proportions (prevalence) meta-analysis was conducted using the Freeman–Tukey double arcsine transformation, the DerSimonian–Laird estimator for the between-study variance, tau^2^, and the Jackson method for the confidence intervals for tau^2^ and tau. The means (mean distances) meta-analysis was conducted using the untransformed (raw) means, the restricted maximum-likelihood estimator for tau^2^, and the Q-Profile method for the confidence intervals for tau^2^ and tau. Moreover, several subgroup analyses were performed to detect variables (geographic distribution, sample size, and study type) affecting the estimated pooled prevalence and pooled mean. A *p*-value of less than 0.05 was considered statistically significant. Cochran’s Q statistic was used to evaluate the presence of heterogeneity across studies, and the Higgins I^2^ statistic was used to quantify heterogeneity. Cochran’s Q *p*-value < 0.10 was considered significant. Higgins I^2^ values between 0 and 40% were regarded as low heterogeneity, between 30% and 60% as moderate heterogeneity, between 50% and 90% as substantial heterogeneity, and between 75% and 100% as considerable heterogeneity. To evaluate the presence of the small-study effect (the phenomenon that smaller studies may show different effects than large ones), a DOI plot with an LFK index was generated for proportional parameters [27], and a funnel plot was generated with the Thompson–Sharp test for continuous parameters [28].

## 3. Results

**Figure 1:**
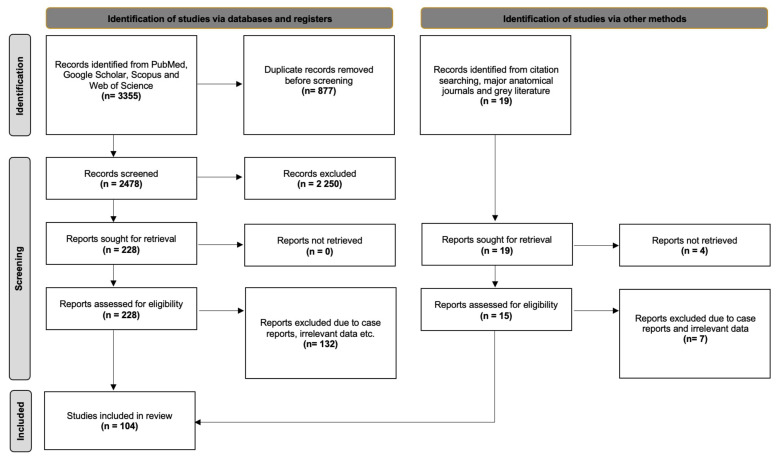
Flow chart of the literature search per PRISMA 2020 guidelines [23].

The database search identified 3355 articles exported to Mendeley version 2.10.9 (Elsevier, London, UK). After excluding duplicate and irrelevant papers (title and abstract screening), 228 studies underwent full-text retrieval and screening. Finally, 96 studies were eligible for the systematic review. Furthermore, 19 studies were identified from our secondary investigation (manual search of references, grey literature, and anatomical journals). Thus, eight studies were included in our systematic review with meta-analysis from our secondary investigation. Figure 1 presents a flow diagram of our search analysis based on the PRISMA 2020 guidelines.

One hundred and four (104) studies were included, with a total sample of 136,010 heminecks. Eighty-six (86) papers were imaging studies, and nineteen (19) were osteological studies. The mean sample per article was 1295.33 heminecks. Fifty-seven (57) studies belonged to the Asian population, twenty-five (25) to the European population, twenty-one (21) to the American population, one (1) to the African population, and one (1) to the Oceanian population. The characteristics of the included studies are summarized in Table 1.

The typical SP (length under 30 mm) was calculated with an overall pooled prevalence of 74.97% (95% CI: 71.59–79.35). Its bilateral appearance was estimated with a pooled prevalence of 72.66% (95% CI: 66.36–78.54). The subgroup analysis is presented in Table 2. A significant association was identified based on the study type. The typical SP pooled prevalence was higher in the osteological studies (85.17%) and lower in the computed tomography (CT) studies (69.33%) (*p* = 0.0042). The DOI plot retrieved an LFK index of −2.67 (significant asymmetry), indicating a small study effect on the pooled prevalence.

An elongated SP was identified when its length was more than 30 mm. However, seventeen (17) studies considered a different threshold (25 or 40, or 33 mm). The elongated SP was estimated to have a pooled prevalence of 25.03% (95% CI: 21.23–29.03). Its bilateral appearance was identified with a pooled prevalence of 16.04% (95% CI: 12.00–20.54). The bilateral asymmetrical morphology (typical and elongated SP in the same patient) was estimated to have a pooled prevalence of 9.22% (95% CI: 7.16–11.51). The subgroup analysis is summarized in Table 2. A significant association was identified based on the study’s type. The osteological studies reported a lower prevalence of elongated SP (14.83%), and the CT studies reported the highest prevalence (30.67%). The DOI plot depicted an LFK index of +0.38 (no asymmetry), indicating no small-study effect on the pooled prevalence.

The SP length was calculated to have a pooled mean of 28.91 mm (95% CI: 17.18–30.64). The subgroup analysis is presented in Table 3. Statistically significant differences were observed based on the geographical distribution (*p* = 0.0035) and study type (*p* = 0.0029). The Thompson–Sharp test results indicate no funnel plot asymmetry for the pooled mean length (*p* = 0.2033).

**Figure 2:**
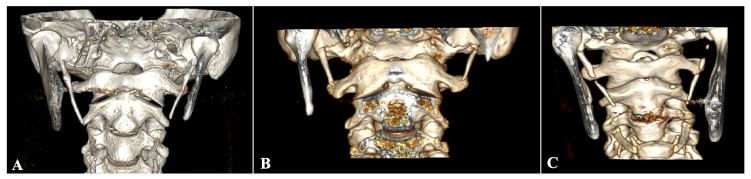
(**A**) Bilateral elongated styloid processes (SPs), (**B**) bilateral typical SP, (**C**) bilateral variable ossification of the stylohyoid chain.

Several studies have classified SHC ossification according to Langlais et al. [129] (Figure 2). Langlais Type I (elongated SP in a unique segment) was estimated to have a pooled prevalence of 16.35% (95% CI: 11.36–22.04). Langlais Type II (pseudoarticulated SP—partially calcified) was estimated to have a pooled prevalence of 4.39% (95% CI: 2.58–6.64). Langlais Type III (segmented SP—completely calcified) was observed to have a pooled prevalence of 3.89% (95% CI: 2.62–5.39). The subgroup analysis is summarized in Table 4. Significant differences were observed based on the geographical distribution and study type.

## 4. Discussion

The present systematic review with meta-analysis evaluated the SHC typical anatomy and possible morphological variants, including SP length and SHC variable ossification. We found that imaging techniques are more reliable than osteological ones because they can also identify SHC variable ossification, which is typically present in segments. Nevertheless, SP elongation can be derived from extensive SHC ossification with articulation. Therefore, researchers should investigate the entire SHC anatomy and not only the SP length.

Typical SPs were identified in 74.97% of cases, with a mean length of 28.91 mm. Elongated SPs were observed in 25.03%. Although elongated SPs are considered to be over 30 mm, several studies have considered a different threshold, with 25 mm or 40 mm being the most commonly reported. Jung et al. [73] and Natsis et al. [10] proposed that SP elongation should be considered after statistical analysis of the sample’s percentiles. Natsis et al. [10] considered the 25–75th percentiles as the standard length; therefore, they reported a 33 mm threshold. This method proposed by Jung et al. [73] and Natsis et al. [10] should be carefully considered by future researchers because every sample is unique, and there may be variations in the geographical distribution of SP length. Nevertheless, we identified substantial differences between the study types. In the previous meta-analysis of only imaging studies, a pooled prevalence of 30.2% for SP elongation was calculated by Noguira-Reis et al. [21]. Several researchers have indicated that imaging technique is essential for elongated SP identification. For example, in a CT scan with three-dimensional reconstruction (3D), the accurate length can be calculated based on the prominent anatomical landmarks (the base of the external acoustic meatus and point of SP emersion from the temporal bone and the SP tip) [128]. This is why 3DCT is the best option [3]. The influence of age on SP length is controversial in the current literature. However, only a few studies have analyzed the age effect [42,66,74,126]. These studies have observed that patients under 20 years old had a lower prevalence of SP elongation. Contrariwise, the survey by Natsis et al. [10] found no age effect.

SHC ossification was classified by most of the studies according to the method Langlais et al. [129]. Although this was the first classification method based on 4200 X-rays, new techniques (CT or cone beam CT (CBCT) scans) are better for visualizing the SHC. Andrei et al. [33] performed a detailed analysis of the SP and SHC based on CBCT. They classified their results based on the SP length (standard or elongated), angulation, shape, variable number of its segments (one, two, or three pieces) separated by a pseudoarthrosis, and variable ossification degree (complete or incomplete). The classification method proposed by Andrei et al. [33] seems to be the most complete.

Eagle [1] identified several symptoms associated with an elongated SP, which include persistent pain in the pharynx, ear pain from irritation of the vagus nerve, increased salivation, difficulty swallowing, and the sensation of having a foreign body in the throat. He noted that these symptoms typically emerged following a tonsillectomy, likely due to scar tissue formation. Additionally, Eagle highlighted another group of symptoms related to carotid artery syndrome and proposed that surgical intervention is the most effective treatment for SP elongation, often yielding excellent results.

An elongated SP can affect both the ICA and the ECA in the context of stylocarotid artery syndrome. Stimulation of the ICA can cause pain along its pathway and parietal cephalalgia, while ECA irritation may result in facial pain and contribute to atherosclerosis due to increased mechanical pressure [14]. Recent studies have explored the relationship between the elongated SP and the ICA-ECA. Triantafyllou et al. [5] discovered that both arteries were positioned closer to an elongated SP, with the ICA being nearer when the SHC was variably ossified. They documented three distinct topographical patterns regarding the spatial relationship between the SP and these vascular structures. The ECA was laterally adjacent in 80% of cases, while the ICA was medial to the SP. In 14.2% of cases, the ECA was situated anterolaterally and the ICA anteromedially relative to the SP. The least common arrangement, observed in 5.8% of cases, had both the ECA and ICA positioned posteriorly to the SP. Calota et al. [4] reported that in 11.88% of specimens with an elongated SP, the ECA took a retrostyloid course, entering the parapharyngeal space, which can present surgical challenges. Furthermore, an elongated SP has been linked to temporomandibular joint disorders.

The current meta-analysis has several limitations. Firstly, we observed a significant degree of heterogeneity and an elevated risk of bias among the studies, a common issue in anatomical meta-analyses [22]. Furthermore, the pooled prevalence for the typical SP exhibited considerable asymmetry in the DOI plot, indicating a potential small-study effect [27]. We could not conduct subgroup analyses by age group, particularly for children, due to a lack of sufficient data in the existing literature. Lastly, a few subgroup analyses did not meet the minimum requirement of four studies, which is generally deemed necessary for drawing reliable conclusions.

## 5. Conclusions

The anatomy of the SHC has been re-evaluated in the current evidence-based systematic review accompanied by a meta-analysis. A typical SP, under 30 mm and without ossification on the SHC, was identified in 74.97% of cases, while elongated SPs were present in 25.03%. Both morphologies are statistically more common bilaterally. This highlights the significant variability in SP anatomy. Additionally, the analysis indicated a statistically significant difference among various imaging studies, suggesting that CT with three-dimensional reconstruction should be considered the gold standard for visualizing typical anatomy and possible variants of the SP and SHC. A thorough understanding of these variants and their clinical implications is crucial for anatomists, radiologists, and clinicians.

## Figures and Tables

**Table 1:** The characteristics of the included studies according to the year, the study’s geographic origin, the study type, the sample, the age group, and the risk of bias according to the AQUA tool by Henry et al. [26].

Study	Year	Population	Study Type	Size	Age Group	Risk of Bias
Altay et al. [29]	2024	Asia	X-ray	6024	Adults and children	High
Alzaera [30]	2017	Asia	X-ray	396	Adults	High
Amorim et al. [31]	2017	Europe	CT	124	Adults	High
Anbiaee et al. [32]	2011	Asia	X-ray	207	Adults and children	High
Andrei et al. [33]	2013	Europe	CT	88	Adults	Low
Aoun et al. [34]	2020	Asia	X-ray	978	Adults	Low
Assiri Ahmed et al. [35]	2023	Europe	X-ray	800	Adults	High
Baena-Caldas et al. [36]	2017	America	Osteological	46	NR	High
Bagga et al. [37]	2012	Asia	X-ray	5412	Adults	Low
Bagga et al. [38]	2021	Asia	X-ray	3412	Adults	Low
Balcioglu et al. [39]	2009	Asia	X-ray and osteological	495	Adults	High
Basekim et al. [3]	2005	Asia	CT	269	Adults and children	High
Baykann et al. [40]	2019	Asia	CT	154	Adults	High
Bozkir et al. [41]	1999	Asia	X-ray	400	Adults	High
Bruno et al. [42]	2017	Europe	CT	2006	Adults and children	Low
Buyuk et al. [43]	2017	Asia	CT	2000	Adults	High
Camarda et al. [44]	1989	America	X-ray	300	Adults and children	High
Castro-Espinoza et al. [45]	2020	America	X-ray	4050	Adults and children	Low
Cavalcante et al. [46]	2017	America	X-ray	1890	Adults and children	High
Chu et al. [47]	2022	Asia	CT	230	Adults	High
Correll et al. [48]	1979	America	X-ray	3542	Adults	Low
Costantinides et al. [49]	2019	Europe	X-ray	320	Adults	High
Cullu et al. [50]	2013	Asia	CT	320	Adults	High
Custodio et al. [51]	2016	America	Osteological	30	NR	High
De Cosra et al. [52]	2014	America	CT	342	Adults	High
De Paz et al. [53]	2012	Europe	Osteological		NR	High
Domnez et al. [54]	2017	Asia	CT	2000	Adults and children	Low
Dos Santos Accioly Lins et al. [55]	2015	America	X-ray	1120	Adults	High
Dudde et al. [56]	2024	Europe	CT	200	Adults	Low
Ekici et al. [57]	2013	Asia	MDCT	1610	Adults	Low
Eraslan et al. [58]	2017	Asia	CT	250	Adults	High
Ferrario et al. [59]	1990	Europe	X-ray	572	Adults and children	Low
Frommer [18]	1974	America	Osteological	241	Adults	High
Garay et al. [60]	2013	America	X-ray	6056	Adults and children	High
Ghafari et al. [61]	2012	Asia	X-ray	592	Adults	Low
Ghassemzahed et al. [62]	2021	Europe	X-ray	4614	Adults and children	Low
Gokce et al. [63]	2008	Asia	X-ray	1396	Adults and children	High
Gomes do Nascimento Junior [64]	2015	America	X-ray	600	Adults and children	High
Gozil et al. [65]	2001	Asia	X-ray	210	Adults	High
Gracco et al. [66]	2016	Europe	X-ray	1200	Adults and children	Low
Guimaraes et al.	2020	America	X-ray	8826	Adults	High
Guo et al. [67]	2014	Asia	CT	100	Adults and children	High
Hettiarachchi et al. [68]	2019	Asia	X-ray	185	Adults	Low
Ilguy et al. [69]	2005	Asia	X-ray	1720	Adults	High
Ilguy et al. [70]	2013	Asia	CBCT	138	Adults and children	Low
Jeevitha et al. [71]	2023	Asia	X-ray	800	Adults	Low
Joshi et al. [72]	2007	Asia	X-ray	106	Adults and children	High
Jung et al. [73]	2004	Europe	X-ray	837	Adults and children	High
Kaaki et al. [74]	2024	Asia	X-ray	745	Adults and children	High
Kapur et al. [75]	2022	Europe	Osteological	400	Adults	Low
Kaufman et al. [2]	1970	America	X-ray	968	Adults	High
Keur et al. [76]	1986	America	X-ray	2270	Adults	Low
Kevin O Carroll et al. [77]	1984	America	X-ray	958	Adults and children	High
Koshy et al. [78]	2014	Asia	Osteological	90	NR	High
Kursoglu et al. [79]	2005	Asia	X-ray	110	Adults	High
Krenmair et al. [80]	2003	Europe	X-ray	795	Adults	High
Kurbanova et al. [81]	2024	Europe	CBCT	743	Adults	Low
Lengele et al. [82]	1988	Europe	Osteological	404	Adults	Low
Magat et al. [83]	2017	Asia	X-ray	1820	Adults	Low
Margam et al. [84]	2015	Asia	Osteological	140	NR	High
McDonald-Jankowski [85]	2001	Europe & Asia	X-ray	3324	NR	High
Missias et al. [86]	2017	America	CBCT	2000	Adults	High
Monsour et al. [87]	1986	Australia	X-ray	670	Adults and children	High
More and Arsani [88]	2010	Asia	X-ray	1000	Adults	High
Muneera et al. [89]	2021	Asia	Osteological	142	Adults	High
Munoz-Leija et al. [90]	2020	America	CT	198	Adults	High
Natsis et al. [10]	2015	Europe	Osteological	262	Adults	Low
Nemanic et al. [91]	2009	Europe	Osteological	122	Adults and children	High
Okabe et al. [92]	2006	Asia	X-ray	1318	Adults (only 80 years)	Low
Onbas et al. [93]	2005	Asia	X-ray	566	Adults	Low
Oztas et al. [94]	2012	Asia	X-ray	4000	Adults and children	High
Paraskevas et al. [95]	2022	Asia	Osteological	127	Adults	High
Patil et al. [96]	2014	Europe	Osteological	228	NR	High
Phulambrikar et al. [97]	2011	Asia	X-ray	328	Adults and children	High
Ramadan et al. [98]	2007	Asia	CT	200	Adults	Low
Rath and Anand [99]	1991	Asia	Osteological	464	NR	High
Rathva et al. [100]	2013	Asia	Osteological	300	NR	High
Reddy et al. [101]	2013	Asia	X-ray	520	Adults and children	High
Ribeiro et al. [102]	2018	Europe	X-ray	4750	Adults and children	Low
Rizzatti-Barbosa et al. [103]	2005	America	X-ray	4504	Adults	High
Roopashri et al. [104]	2012	Asia	X-ray	600	Adults and children	High
Ruprecht et al. [105]	1988	Asia	X-ray	1042	Adults and children	High
Saati et al. [106]	2020	Asia	X-ray	4054	Adults	Low
Safabakhsh et al. [107]	2018	Asia	X-ray	10,000	Adults and children	High
Sahed et al. [108]	2011	Asia	Osteological	1188	Adults	High
Sakhadari et al. [109]	2018	Asia	X-ray	1000	Adults	Low
Saric et al. [110]	2023	Europe	CT	1658	Adults	Low
Scaf et al. [111]	2003	America	X-ray	332	NR	High
Shah et al. [112]	2012	Asia	X-ray	1034	NR	High
Shahidi et al. [113]	2021	Asia	CBCT	698	Adults and children	Low
Sharma et al. [114]	2019	Asia	X-ray	2000	Adults and children	High
Shayganfar et al. [115]	2018	Asia	MDCT	786	Adults and children	High
Smit et al. [116]	2019	Africa and Europe	X-ray and osteological	45	NR	High
Sokler and Sandev [117]	2001	Europe	X-ray	308	Adults and children	High
Srivedi et al. [118]	2019	Asia	X-ray	1000	Adults	Low
Swapna et al. [119]	2021	Asia	X-ray	600	Adults and children	High
Tanaka et al. [120]	2022	Asia	Osteological	78	NR	High
Tavares et al. [121]	2007	America	X-ray	926	Adults and children	Low
Tiwary et al. [122]	2017	Asia	X-ray	168	Adults and children	Low
Togan et al. [123]	2016	Europe	CBCT	1998	Adults and children	Low
Vadgaonkar et al. [124]	2015	Asia	Osteological	220	NR	High
Vasilopoulos et al. [125]	2021	Europe	Osteological	363	Adults	Low
Vieiera et al. [126]	2015	America	X-ray	1472	Adults and children	Low
Zang et al. [127]	2020	Asia	CT	156	Adults	High
Zokaris et al. [128]	2019	Europe	OPG	1610	Adults and children	Low

**Table 2:** The results of the normal and elongated styloid process (SP). k represents the number of studies. Statistically significant results are presented with an asterisk.

Parameters	Typical SP (%)	Elongated SP (%)
Overall Prevalence (k = 79)	74.97	25.03
Bilateral Prevalence (k = 38)	72.66	16.04
Asia (k = 39)	77.35	22.66
Europe (k = 18)	74.92	26.80
Africa (k = 3)	79.45	27.01
America (k = 18)	71.54	28.46
Oceania (k = 1)	78.96	21.04
*p*-Value	*p *= 0.5310	*p *= 0.3782
X-ray (k = 50)	74.19	26.78
CT (k = 14)	69.33	30.67
Osteological (k = 11)	85.17	14.83
CBCT (k = 4)	84.92	15.08
*p*-Value	*p *= 0.0042 *	*p *= 0.0032 *
Male (k = 28)	69.24	30.76
Female (k = 28)	73.30	25.86
*p*-Value	*p *= 0.4872	*p *= 0.3977
Left (k = 41)	78.77	21.91
Right (k = 41)	78.82	21.85
*p*-Value	*p *= 0.9903	*p *= 0.9866

**Table 3:** The pooled mean length of the styloid process (SP). k represents the number of studies. Statistically significant results are presented with an asterisk.

Parameters	SP Pooled Mean Length (in mm)
Overall Prevalence (k = 27)	28.81
Asia (k = 19)	27.71
Europe (k = 6)	31.81
Africa (k = 0)	-
America (k = 1)	35.10
Oceania (k = 1)	29.20
*p*-Value	*p *= 0.0035 *
X-ray (k = 8)	30.31
CT (k = 12)	29.87
Osteological (k = 6)	25.60
CBCT (k = 1)	25.30
*p*-Value	*p *= 0.0029 *
Male (k = 19)	29.83
Female (k = 19)	27.61
*p*-Value	*p *= 0.1821
Left (k = 31)	27.45
Right (k = 31)	27.27
*p*-Value	*p *= 0.9666

**Table 4:** The stylohyoid chain (SHC) ossification pattern, according to Langlais et al.’s (1986) [129] classification system. k represents the number of studies. Statistically significant results are presented with an asterisk.

Parameters	Type 1 SHC (%)	Type 2 SHC (%)	Type 3 SHC (%)
Overall Prevalence (k = 18)	16.35	4.39	3.89
Asia (k = 12)	13.67	3.97	3.36
Europe (k = 0)	-	-	-
Africa (k = 2)	10.09	3.12%	1.45
America (k = 4)	29.08	6.55	7.43
Oceania (k = 0)	-	-	-
*p*-Value	*p* < 0.0001 *	*p* = 0.7532	*p* < 0.0001 *
X-ray (k = 14)	17.47	3.01	3.12
CT (k = 4)	12.74	11.59	7.61
Osteological (k = 0)	-	-	-
CBCT (k = 0)	-	-	-
*p*-Value	*p* = 0.2716	*p* = 0.0550	*p* = 0.0061 *
Male (k = 5)	30.36	9.03	4.28
Female (k = 5)	24.09	9.09	6.53
*p*-Value	*p* = 0.1785	*p* = 0.9616	*p* = 0.1804
Left (k = 14)	22.74	4.38	3.62
Right (k = 14)	20.99	4.84	4.06
*p*-Value	*p* = 0.6948	*p *= 0.8009	*p* = 0.6974

## Data Availability

The data are available upon reasonable request to the corresponding authors (Maria Piagkou—mapian@med.uoa.gr).

## Supplementary Materials

The following supporting information can be downloaded at: https://www.mdpi.com/article/10.3390/diagnostics15070925/s1, Supplementary Table S1. The detailed Anatomical Quality Assurance (AQUA) tool of the included studies. Each domain has four to six questions that should be answered for each included study. The possible answers are “Yes”, “No”, or “Unclear”, which are translated into “Low”, “High”, or “Unclear” risk of bias. If (at least) one domain is considered as having “High” risk of bias, the included study should be considered as having “High” risk of bias. A study could be considered as having “Low” risk of bias when all five domains are “Low”.

## Abbreviations

The following abbreviations are used in this manuscript:

SP	Styloid process
SHC	Stylohyoid chain
SHL	Stylohyoid ligament
HB	Hyoid bone
LH	Lesser horn
ECA	External carotid artery
ICA	Internal carotid artery
CT	Computed tomography
CBCT	Come bean computed tomography
