# Supplementary material for: The Anatomy of the Stylohyoid Chain: A Systematic Review with Meta-Analysis
Source: Diagnostics (Basel). 2025 Apr 3;15(7):925. doi: 10.3390/diagnostics15070925 (PMC11988981; doi:10.3390/diagnostics15070925)
Supplement: Supplementary file 1 [file diagnostics-15-00925-s001.zip › diagnostics-3491580-supplementary.pdf]

Supplementary Table S1. The detailed Anatomical Quality Assurance (AQUA) tool of the included studies. Each domain has four to six questions that it should be answered for each included study. The possible answers are "Yes", "No" or "Unclear" that are translated to "Low", "High" or "Unclear" risk of bias. If (at least) one domain is considered "High" risk of bias, the included study should be considered "High" risk of bias. A study could be considered "Low" risk of bias when all five domains are "Low".

Study	Domain 1: Objectives	Domain 2: Design	Domain 3: Methodology	Domain 4: Descriptive	Domain 5: Results Report
Altay et al. [29]	High	Low	Low	Low	Low
Alzaera [30]	High	Low	High	Low	Low
Amorim et al. [31]	High	Low	Low	Low	Low
Anbiaee et al. [32]	Low	Low	High	Low	Low
Andrei et al. [33]	Low	Low	Low	Low	Low
Aoun et al. [34]	Low	Low	Low	Low	Low
Assiri Ahmed et al. [35]	High	Low	Low	Low	High
Baena-Caldas et al. [36]	High	Low	High	Low	Low
Bagga et al. [37]	Low	Low	Low	Low	Low
Bagga et al. [38]	Low	Low	Low	Low	Low
Balcioglu et al. [39]	High	Low	High	Low	Low
Basekim et al. [3]	High	Low	Low	Low	Low
Baykann et al. [40]	Low	Low	Low	High	Low
Bozkir et al. [41]	High	Low	High	Low	High
Bruno et al. [42]	Low	Low	Low	Low	Low
Buyuk et al. [43]	High	Low	Low	Low	Low
Camarda et al. [44]	High	Low	High	Low	Low
Castro-Espinoza et al. [45]	Low	Low	Low	Low	Low
Cavalcante et al. [46]	Low	Low	High	Low	Low
Chu et al. [47]	High	Low	Low	Low	Low
Correll et al. [48]	Low	Low	Low	Low	Low
Costantinides et al. [49]	Low	Low	High	Low	High
Cullu et al. [50]	Low	Low	High	Low	High
Custodio et al. [51]	High	Low	High	Low	Low
De Cosra et al. [52]	High	Low	High	Low	Low
De Paz et al. [53]	Low	Low	Low	High	Low
Domnez et al. [54]	Low	Low	Low	Low	Low
Dos Santos Accioly Lins et al. [55]	Low	High	Low	Low	Low
Dudde et al. [56]	Low				
Ekici et al. [57]	Low	Low	Low	Low	Low

Eraslan et al. [58]					
Ferrario et al. [59]	Low	Low	Low	Low	Low
Frommer [18]	High	Low	High	Low	Low
Garay et al. [60]	Low	Low	High	Low	Low
Ghafari et al. [61]	Low	Low	Low	Low	Low
Ghassemzahed et al. [62]	Low	Low	Low	Low	Low
Gokce et al. [63]	Low	Low	Low	High	Low
Gomes do Nascimento Junior [64]	High	Low	Low	High	Low
Gozil et al. [65]	Low	Low	High	Low	Low
Gracco et al. [66]	Low	Low	Low	Low	Low
Guimaraes et al.	Low	Low	High	Low	Low
Guo et al. [67]	Low	Low	High	Low	Low
Hettiarachchi et al. [68]	Low	Low	Low	Low	Low
Ilguy et al. [69]	High	Low	Low	Low	Low
Ilguy et al. [70]	Low	Low	Low	Low	Low
Jeevitha et al. [71]	Low	Low	Low	Low	Low
Joshi et al. [72]	Low	Low	Low	High	Low
Jung et al. [73]	High	Low	Low	Low	Low
Kaaki et al. [74]	High	Low	High	High	Low
Kapur et al. [75]	Low	Low	Low	Low	Low
Kaufman et al. [76]	High	Low	Low	High	Low
Keur et al. [77]	Low	Low	Low	Low	Low
Kevin O Carroll et al. [78]	High	Low	High	High	Low
Koshy et al. [79]	High	Low	High	High	High
Kursoglu et al. [80]	Low	Low	Low	High	Low
Krenmair et al. [81]	Low	Low	High	High	Low
Kurbanova et al. [82]	Low	Low	Low	Low	Low
Lengele et al. [83]	Low	Low	Low	Low	Low
Magat et al. [84]	Low	Low	Low	Low	Low
Margam et al. [85]	High	Low	Low	Low	Low
McDonald-Jankowski [86]	High	Low	High	High	Low
Missias et al. [87]	Low	High	Low	Low	Low
Monsour et al. [88]	High	Low	High	High	Low
More and Arsani [89]	High	Low	Low	High	Low
Muneera et al. [90]	High	Low	High	Low	Low
Munoz-Leija et al. [91]	Low	High	Low	Low	Low
Natsis et al. [10]	Low	Low	Low	Low	Low
Nemanic et al. [92]	High	Low	Low	Low	Low

Okabe et al. [93]	Low	Low	Low	Low	Low
Onbas et al. [94]	Low	Low	Low	Low	Low
Oztas et al. [95]	Low	Low	High	Low	Low
Paraskevas et al. [96]	Low	Low	High	Low	Low
Patil et al. [97]	High	Low	High	Low	Low
Phulambrikar et al. [98]	High	Low	Low	High	Low
Ramadan et al. [99]	Low	Low	Low	Low	Low
Rath and Anand [100]	High	Low	High	Low	Low
Rathva et al. [101]	High	Low	High	Low	Low
Reddy et al. [102]	High	Low	Low	Low	Low
Ribeiro et al. [103]	Low	Low	Low	Low	Low
Rizzatti-Barbosa et al. [104]	Low	Low	Low	Low	High
Roopashri et al. [105]	Low	Low	High	Low	Low
Ruprecht et al. [106]	High	Low	Low	High	Low
Saati et al. [107]	Low	Low	Low	Low	Low
Safabakhsh et al. [108]	Low	Low	Low	High	Low
Sahed et al. [109]	High	Low	High	High	Low
Sakhadari et al. [110]	Low	Low	Low	Low	Low
Saric et al. [111]	Low	Low	Low	Low	Low
Scaf et al. [112]	High	Low	High	Low	High
Shah et al. [113]	High	Low	High	Low	Low
Shahidi et al. [114]	Low	Low	Low	Low	Low
Sharma et al. [115]	Low	Low	Low	High	Low
Shayganfar et al. [116]	Low	High	Low	High	Low
Smit et al. [117]	High	Low	High	Low	Low
Sokler and Sandev [118]	High	Low	High	High	High
Srivedi et al. [119]	Low	Low	Low	Low	Low
Swapna et al. [120]	Low	Low	High	High	Low
Tanaka et al. [121]	Low	High	Low	Low	Low
Tavares et al. [122]	Low	Low	Low	Low	Low
Tiwary et al. [123]	Low	Low	Low	Low	Low
Togan et al. [124]	Low	Low	Low	Low	Low
Vadgaonkar et al. [125]	High	Low	Low	Low	Low
Vasilopoulos et al. [126]	Low	Low	Low	Low	Low
Vieiera et al. [127]	Low	Low	Low	Low	Low
Zang et al. [128]	Low	Low	High	Low	Low
Zokaris et al. [129]	Low	Low	Low	Low	Low
